# Impact of surgical approach on complications by sex following ventral and incisional hernia repair

**DOI:** 10.1007/s10029-025-03371-2

**Published:** 2025-05-23

**Authors:** Leah J. Schoel, Joshua Sinamo, Alexander Hallway, Brian T. Fry, John P. Fischer, Sean M. O’Neill, Michael Rubyan, Jenny M. Shao, Dana A. Telem, Anne P. Ehlers

**Affiliations:** 1 Department of Surgery, University of Michigan, 1500 E Medical Center Drive, Ann Arbor, MI 48109 USA; 2 Center for Healthcare Outcomes and Policy, University of Michigan, Ann Arbor, MI USA; 3 Department of Learning Health Sciences, University of Michigan, Ann Arbor, MI USA; 4 Department of Surgery, University of Pennsylvania, Philadelphia, PA USA; 5 Department of Epidemiology, University of Michigan, Ann Arbor, MI USA; 6 Department of Health Management and Policy, University of Michigan, Ann Arbor, MI USA

**Keywords:** Ventral and incisional hernia repair, Hernia care, Outcomes, Sex-based inequity

## Abstract

**Purpose:**

Female patients are more likely than male patients to experience postoperative complications following hernia repair, but the drivers of this phenomenon remain unexplored. Whether surgical approach differentially impacts the likelihood of postoperative complications by sex following ventral and incisional hernia repair (VIHR) remains unknown.

**Methods:**

Adult patients from the Michigan Surgical Quality Collaborative Core Optimization Hernia Registry (MSQC-COHR) were included in this study. MSQC-COHR is a representative, random sample of adult patients from 70 hospitals across Michigan. All elective VIHR performed between January 1, 2020, to September 30, 2023, were included. The primary outcome was any 30-day complication. A multivariable logistics mixed effects model was used to measure the adjusted associations between the observed covariates and the likelihood of 30-day complications. Sex and surgical approach were interacted to test for potential differential effects of surgical approach on 30-day complications by sex.

**Results:**

Among 10,675 patients who underwent elective VIHR, 254 (2.4%) experienced postoperative complications. Of these, 152 (59.8%) patients were female. In bivariate analyses, female patients more often experienced a 30-day complication, regardless of operative approach (3.3% vs. 1.7%, *p* < 0.001). By surgical approach, female patients were found to have 4.2% (95% CI: 3.2–5.1) probability of 30-day complications after open repair, versus 1.5% (95% CI: 1.0-2.1) following MIS VIHR. Male patients had 2.1% (95% CI: 1.5–2.7) probability of 30-day complications after open repair, versus 1.5% (95% CI: 0.9–1.9) following MIS VIHR. Comparatively, female patients were 2.7 times more likely to experience complications after open repair compared to MIS repair, while male patients were 1.4 times more likely to have complications after open vs. MIS repair.

**Conclusion:**

Following both open and MIS VIHR, female patients have a higher risk of postoperative complications compared to male patients, with this risk increasing after open repairs. The outcome disparity persists even after accounting for differences in comorbidities and hernia characteristics. Although this sex-based outcome disparity is not fully eliminated, MIS approaches mitigate the gap, suggesting that MIS repairs should be prioritized in female patients when feasible.

## Introduction

It is well-established that females fare worse following many common operations [1–3]. Nowhere is this more true than in abdominal wall hernia repair, where multiple prior studies have demonstrated worse clinical and patient-reported outcomes for female patients [4–7]. Female patients consistently demonstrate higher rates of postoperative complications, including surgical site infections, readmission, and recurrence [8, 9]. A previous study assessing sex disparities in outcomes following ventral and incisional hernia repairs (VIHR) found that female patients had an aggregated 11.7% predicted probability of adverse events, compared to 7.6% in male patients [5]. Similarly, female patients report higher rates of postoperative pain than males [10–17]. Female patients are also more likely than males to express decision regret—remorse for having undergone the surgical procedure—following hernia repair [18, 19]. Identifying specific and intervenable factors that contribute to these poor outcomes among female patients may lead to improved surgical care with more equitable outcomes.

Surgical approach for VIHR is one potentially modifiable aspect that may be associated with differential outcomes between males and females, but to date, the impact of surgical approach remains poorly studied. Existing work has focused specifically on open ventral hernia repair, finding that female patients had higher rates of readmission, wound complications, and interventions for pain as compared to male patients [7]. However, these studies lack comparative analyses across surgical approaches, and whether outcomes vary between open and minimally invasive surgery (MIS) by patient sex remains unknown. The impact of surgical approach on outcomes has been well-described in groin hernia repair, where MIS techniques have been shown to improve outcomes for female patients and mitigate sex disparities in chronic pain and operative recurrence [20–23]. These findings were subsequently incorporated into international guidelines intended to reduce adverse outcomes for female patients after groin hernia repair [24]. Whether surgical approach (e.g., open vs. MIS) differentially impacts outcomes by sex for ventral and incisional hernias remains unknown but may have important implications for improving care for female patients.

Within this context, we sought to evaluate the impact of surgical approach on clinical outcomes between male and female patients following elective ventral and incisional hernia repair. We utilized a novel statewide, population-based hernia registry that prospectively collects clinically nuanced data for persons undergoing hernia repair. We specifically evaluated whether operative approach differentially impacts the short-term (30-day) post-operative outcomes of patients by sex. By understanding whether operative approach impacts outcomes for female patients following VIHR, our study seeks to further guide surgical management, refine pre-operative counseling, and improve shared decision-making [22–24].

## Materials and methods

### Data source

Data were analyzed from the Michigan Surgical Quality Collaborative Core Optimization Hernia Registry (MSQC-COHR). The MSQC is a statewide quality improvement program in Michigan that prospectively collects patient demographic, clinical, and outcome data from member hospitals with the aid of professionally trained clinical nurse abstractors [25, 26]. As previously described, the cases in the registry are sampled using an algorithm designed to minimize selection bias and to capture a representative sample of all major operations performed in Michigan [25]. The registry performs regular inter-rater reliability assessments to ensure the validity and reliability of data, and all data are audited annually for accuracy. To obtain specific operative details, data abstraction involves review of the surgical note and items or devices billed in the operating room. The MSQC is classified as a Patient Safety Organization by the Agency for Healthcare Research and Quality, which recognizes the highest level of quality and security in the methods by which data are gathered, analyzed, and shared by each site [26].

The MSQC-COHR is a hernia-specific registry involving 70 hospitals across Michigan that began collection of hernia-specific data in January 2020. In addition to the variables captured in the MSQC, the MSQC-COHR prospectively collects detailed hernia- and operative-specific data, such as hernia defect size and location, operative approach (open, laparoscopic, robotic), mesh use, mesh type, mesh location, and use of advanced techniques [25]. A list of hernia-specific variables can be found in Table 1.

**Table 1:** Hernia-specific variables collected in the MSQC database

Data Element	Details
Hernia location	Epigastric, umbilical, infraumbilical, suprapubic, no midline component
Initial or recurrent	Did the patient have a previous hernia repair?
Hernia width	Width in cm
Hernia length	Length in cm
Mesh placement	Was mesh used?
Mesh width	Width in cm
Mesh length	Length in cm
Type of mesh	Synthetic nonabsorbable, synthetic absorbable, biosynthetic, biologic, other
Mesh brand	Brand of mesh (e.g., Bard, Medtronic, Ethicon, etc.)
Mesh location	Onlay, inlay, sublay, unknown
Mesh fixation	Suture, adhesive, absorbable tacks, non-absorbable tacks, self-fixating, other/multiple

### Patient population

This study included all adult patients aged 18 or older who underwent elective VIHR in MSQC-COHR from January 1, 2020, to September 30, 2023. This sample included cases with the following CPT codes: 49,560, 49,561, 49,565, 49,566, 49,570, 49,572, 49,585, 49,587, 49,590, 49,652, 49,653, 49,654, 49,655, 49,656, 49,657.

Prior to analysis, we excluded cases performed at hospitals with less than 10 total cases recorded across the entire study period. This exclusion was intended to ensure adequate sample size by site given that our analysis included site as a random intercept, and a small sample size by site would skew the analysis. This resulted in 9 cases excluded from 2 hospitals. Further, we excluded cases with hernia sizes greater than the 99th percentile (> 17 cm), which resulted in exclusion of 112 additional cases. This exclusion was intended to confirm that the analyses encapsulated cases and patterns for more typical-sized hernias. A total of 121 cases were excluded prior to analysis.

### Explanatory variables & outcomes

We focused on the interactive effects between sex and surgical approach. Surgical approach was categorized as open or minimally invasive (MIS), with MIS including both laparoscopic and robotic techniques.

We assessed the relationship between the primary outcome (30-day complications) and the following explanatory variables: age, sex (male/female), race (single race White, single race Black, other single or multi-racial), Hispanic ethnicity, insurance type (commercial, non-commercial), past-year cigarette smoking; pre-operative: morbid obesity (BMI > 40), diabetes (no diabetes, diabetes managed without insulin, diabetes managed with insulin), congestive heart failure, hypertension, chronic condition requiring steroids, deep vein thrombosis, ascites, ventilator use, cancer, dialysis, sleep apnea, chronic obstructive pulmonary disease (COPD); surgical and hernia specific: approach (open, minimally invasive surgery (MIS)), hernia size (width or diameter), hernia location (umbilical, epigastric, infraumbilical, suprapubic, no midline component), history of previous hernia repair, the use of mesh, mesh location, and ASA classification (1–4).

The primary outcome measure was 30-day complications. The variable 30-day complications was constructed to denote the occurrence of any of the following 30-day complications: superficial incisional surgical space infection (SSI); deep incisional SSI; organ/space SSI; pneumonia; unplanned intubation– intraoperative; unplanned intubation– postoperative; pulmonary embolism; acute kidney injury; urinary tract infection (UTI) - non-catheter-associated urinary tract infections (CAUTI); UTI– CAUTI; stroke/cerebral vascular accident (CVA); cardiac arrest requiring cardiopulmonary resuscitation (CPR)– intraoperative; cardiac arrest requiring CPR– postoperative; myocardial infarction– intraoperative; myocardial infarction– postoperative; cardiac dysrhythmias; transfusions within first 72 h postoperative; deep vein thrombosis requiring therapy; sepsis; severe sepsis/septic shock; C-difficile; central line-associated bloodstream infection (CLABSI); anastomotic leak; gynecologic - cuff infection; gynecologic - pelvic abscess; gastrointestinal - anastomotic leak; nerve injury - upper extremity; nerve injury - lower extremity; other; septic shock; postoperative ileus requiring nasogastric (NG) tube or non-per-oral (NPO); postoperative urinary retention; or death within 30 days.

### Statistical analysis

Unadjusted, descriptive comparisons were used to describe the observed covariates by sex, followed by the breakdown of the observed covariates by 30-day complication status, using Fisher exact tests for count data. We used a multivariable logistic mixed effect model to measure the adjusted associations between the likelihood of 30-day complications and the observed covariates.

We interacted sex and surgical approach to test for the potential differential effect of surgical approach with respect to 30-day complications by sex. We included hospital sites as random intercepts to allow for the regression model to have varying baseline intercepts, in order to account for case variation between hospital sites. This model both controls for variabilities across sites and similarities of cases within sites by accounting for varying baseline risks across the sites.

This retrospective study was performed in accordance with the ethical standards of the institutional and national research committee, and it was approved by the University of Michigan Institutional Review Board (HUM00091060). All statistical analysis and visualization were performed using R version 4.3.3. All hypotheses were two-sided with α = 0.05. We structured the documentation of this study under the Strengthening the Reporting of Observational Studies in Epidemiology (STROBE) [27].

### Sensitivity analyses

A sensitivity analysis was performed using the same multivariable logistics mixed effect model with the data restricted to participants with typical hernia sizes. Typical hernia sizes were defined as hernias comprising the middle 50% quantile (25th to 75th ) of the overall data, which included hernias between 1.5 cm and 4 cm. Hernias within the 25th to 75th percentile reflect those hernias most commonly seen in clinical practice. This sensitivity analysis was performed to ensure the reliability and validity of the results.

A second sensitivity analysis was performed to evaluate whether surgeon MIS volume was associated with a higher or lower likelihood of 30-day complications by sex. This sensitivity analysis was intended to identify whether the effect of operative approach was persistent across a range of surgeon volume or whether the effect was limited to low-volume or high-volume surgeons. Surgeon-specific MIS volume was quantified as a proportion of total case volume (number of MIS hernia cases/total hernia case volume [open + MIS]). The proportion of MIS case volume was then stratified into tertiles. Tertiles were classified as: low (0th to 33rd percentile), medium (34th to 66th ) and high (67th to 100th ). Surgeons were excluded if their total hernia case volume was less than 10. There were 269 unique surgeons across the state of Michigan that were included. The tertiles were interacted with operative approach and sex to evaluate whether surgeon MIS volume was associated with a higher or lower likelihood of 30-day complications by sex.

### Sub-analysis: laparoscopic versus robotic repairs

A sub-analysis was performed using the same covariates and multivariable logistics mixed effect model from the primary analysis, with the data restricted to MIS repairs. This sub-analysis compared the 30-day complication outcomes by sex between laparoscopic versus robotic repairs. This sub-analysis was intended to identify whether a sex-based difference in outcomes existed between types of MIS repairs.

## Results

### Patient characteristics

A total of 10,675 patients who underwent elective VIHR between 2020 and 2023 were included in this study. Of these, 56.7% were male (*n* = 6050) and 43.3% were female (*n* = 4625) (Table 2). Open approaches were used in 57.1% of all cases (*n* = 6095): 58.7% of cases performed on male patients, and 55.0% of cases performed on female patients. MIS approaches were used in 41.3% of cases performed on male patients, and 45.0% of cases performed on female patients.

**Table 2:** Cohort demographics by sex

	All (*n* = 10675)	Male (*n* = 6050)	Female (*n* = 4625)	*p*-value
Any 30-day complication, n(%)	254 (2.4)	102 (1.7)	152 (3.3)	*p* < 0.001
Age, median (IQR)	55.0 (43–65)	57.0 (46–65)	53.0 (40–64)	*p* < 0.001
Race/Ethnicity, n(%)				*p* < 0.001
White	8965 (84.0)	5301 (87.6)	3664 (79.2)	
Black	1267 (11.9)	504 (8.3)	763 (16.5)	
Other or multi-racial	443 (4.1)	245 (4.0)	198 (4.3)	
Non-commercial insurance, n(%)	6240 (58.5)	3263 (53.9)	2977 (64.4)	*p* < 0.001
Tobacco use, n(%)	2059 (19.3)	1099 (18.2)	960 (20.8)	*p* < 0.001
BMI > 40, n(%)	1414 (13.2)	627 (10.4)	787 (17.0)	*p* < 0.001
ASA 3–4, n(%)	4657 (43.6)	2597 (43.0)	2060 (44.6)	*p* = 0.02
Surgical approach, n(%)				*p* < 0.001
Open	6095 (57.1)	3551 (58.7)	2544 (55.0)	
MIS	4580 (42.9)	2499 (41.3)	2081 (45.0)	
Hernia defect width, cm, median (SD)	2.0 (2.6)	2.0 (2.4)	2.6 (2.9)	*p* < 0.001
Mesh use, n(%)	8362 (78.3)	4782 (79.0)	3580 (77.4)	*p* = 0.04
Mesh location, n(%)				*p* = 0.105
Onlay	1159 (10.9)	651 (10.8)	508 (11)	
Inlay	389 (3.6)	223 (3.7)	166 (3.6)	
Sublay	6421 (60.1)	3699 (61.1)	2722 (58.9)	
Unknown	393 (3.7)	209 (3.5)	184 (4)	
No mesh	2313 (21.7)	1268 (21)	1045 (22.6)	
Previous hernia repair, n(%)	1511 (14.2)	725 (12.0)	786 (17.0)	*p* < 0.001
Hernia location, n(%)				*p* < 0.001
Umbilical	7150 (67.0)	4584 (75.8)	2566 (55.5)	
Epigastric	2297 (21.5)	1029 (17.0)	1268 (27.4)	
Infraumbilical	608 (5.7)	211 (3.5)	397 (8.6)	
Suprapubic	122 (1.1)	31 (0.5)	91 (2.0)	
No midline component	498 (4.7)	195 (3.2)	303 (6.6)	

Overall cohort demographics are available in Table 2. Female patients had larger hernia defects than male patients, with overall median hernia defects of 2.5 cm (SD 2.9) vs. 2.0 cm (SD 2.4) (*p* < 0.001). The majority of hernias were umbilical (67.0%, *n* = 7150), were index repairs (85.8%, *n* = 9164), and used mesh (78.3%, *n* = 8362).

Female patients were more commonly of non-white race (20.8% vs. 12.3%), had non-commercial insurance (64.4% vs. 53.9%), had smoked in the past year (20.8% vs. 18.2%), and had BMI > 40 (17.0% vs. 10.4%) (all *p* < 0.001). Female patients more often had an ASA classification of 3 or 4 compared to male patients (44.6% versus 43.0%, *p* = 0.02), more often had a history of a previous hernia repair (17.0% versus 12.0%, *p* < 0.001), and more often received an MIS approach (45.0% vs. 41.3%, *p* < 0.001), but they less often received mesh during repair (77.4% vs. 79.0%, *p* = 0.04). Complete breakdowns of the covariates by sex are available in Table 2.

Across both sexes, hernias repaired via MIS approaches were larger than those repaired via open approaches. For male patients, mean hernia defect size was 3.2 cm (SD 2.3 cm) for MIS approaches, and 2.5 cm (SD 2.4 cm) for open approaches (*p* < 0.001). For female patients, mean hernia defect size was 3.7 cm (SD 2.7 cm) for MIS approaches, and 3.3 cm (SD 3.1 cm) for open approaches (*p* < 0.001).

### Post-operative outcomes

Of 10,675 patients who underwent elective VIHR, a total of 254 cases (2.4%) were noted to have 30-day complications. Of these 254 cases, 152 (59.8%) were female patients. In bivariate analyses, patients who were female more often had any 30-day complication regardless of operative approach (3.3% versus 1.7%, *p* < 0.001). Additionally, patients who smoked in the past year (*p* < 0.03), had BMI > 40 (*p* < 0.001), had larger hernia sizes (*p* < 0.001), non-umbilical hernias (*p* < 0.001), and/or underwent recurrent hernia repair (*p* < 0.001) were also more likely to have 30-day complications. It was more likely for patients who experienced complications to receive repair via open approach (*p* < 0.001), with the use of mesh (*p* = 0.04), and to have higher ASA classification (*p* < 0.001). Complete breakdowns of the covariates by 30-day complication status are available in Table 3.

**Table 3:** Comparison of cohorts with and without 30-day complications

	All (*n* = 10675)	No 30-day complications (*n* = 10421)	Any 30-day complication (*n* = 254)	*p*-value
Female, n(%)	4625 (43.3)	4473 (42.9)	152 (59.8)	*p* < 0.001
Non-commercial insurance, n(%)	6240 (58.5)	6070 (58.2)	170 (66.9)	*p* = 0.006
Tobacco use, n(%)	2059 (19.3)	1996 (19.2)	63 (24.8)	*p* = 0.03
Morbid obesity, n(%)	1414 (13.2)	1354 (13.0)	60 (23.6)	*p* < 0.001
Surgical approach, n(%)				*p* < 0.001
Open	6095 (57.1)	5916 (56.8)	179 (70.5)	
MIS	4580 (42.9)	4505 (43.2)	75 (29.5)	
Hernia size, cm, n(%)				*p* < 0.001
<2	3418 (32.0)	3381 (32.4)	37 (14.6)	
2-5.9	5920 (55.5)	5815 (55.8)	105 (41.3)	
6+	1337 (12.5)	1225 (11.8)	112 (44.1)	
Previous hernia repair, n(%)	1511 (14.2)	1438 (13.8)	73 (28.7)	*p* < 0.001
Mesh use, n(%)	8362 (78.3)	8150 (78.2)	212 (83.5)	*p* = 0.04
Mesh location, n(%)				*p* = 0.133
Onlay	1159 (10.9)	1124 (10.8)	35 (13.8)	
Inlay	389 (3.6)	376 (3.6)	13 (5.1)	
Sublay	6421 (60.1)	6268 (60.1)	153 (60.2)	
Unknown	393 (3.7)	382 (3.7)	11 (4.3)	
No mesh	2313 (21.7)	2271 (21.8)	42 (16.5)	
Hernia location, n(%)				*p* < 0.001
Umbilical	7150 (67.0)	7019 (67.4)	131 (51.6)	
Epigastric	2297 (21.5)	2216 (21.3)	81 (31.9)	
Infraumbilical	608 (5.7)	586 (5.6)	22 (8.7)	
Suprapubic	122 (1.1)	115 (1.1)	7 (2.8)	
No midline component	498 (4.7)	485 (4.7)	13(5.1)	
ASA Classification, n(%)				*p* < 0.001
1	542 (5.1)	539 (5.2)	3 (1.2)	
2	5476 (51.3)	5398 (51.8)	78 (30.7)	
3	4439 (41.6)	4276 (41.0)	163 (64.2)	
4	218 (2.0)	208 (2.0)	10 (3.9)	

### Differential effect of surgical approach on outcomes by sex

**Fig. 1:**
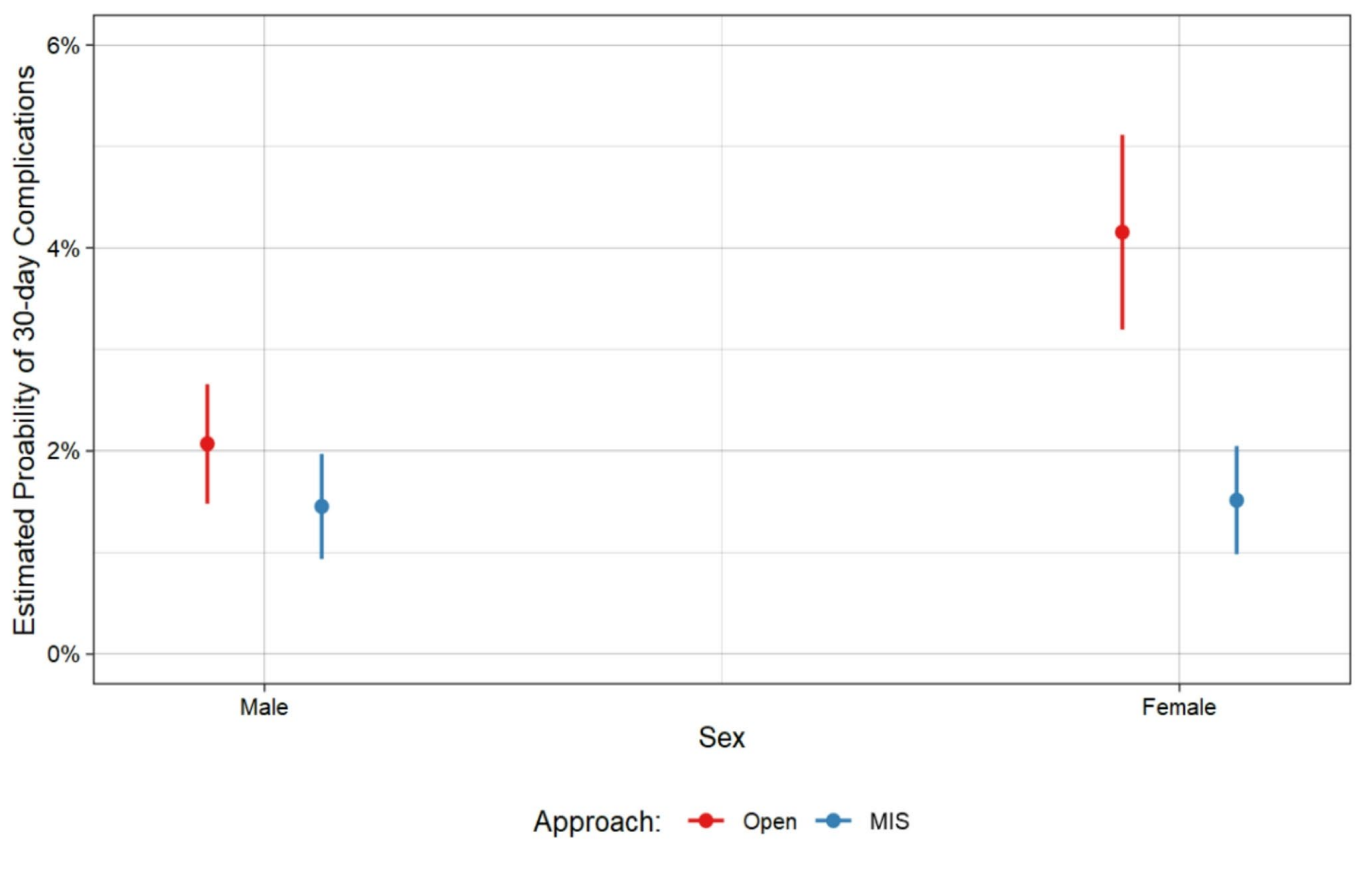
Estimated probability of 30-day complications by sex and surgical approach (*p* < 0.05), all hernia sizes

In a multivariable logistics mixed effects regression model with sites as random intercepts, we found that there was an interactive effect between sex and surgical approach with respect to 30-day complications (*p* < 0.05), as illustrated in Fig. 1.

**Fig. 2:**
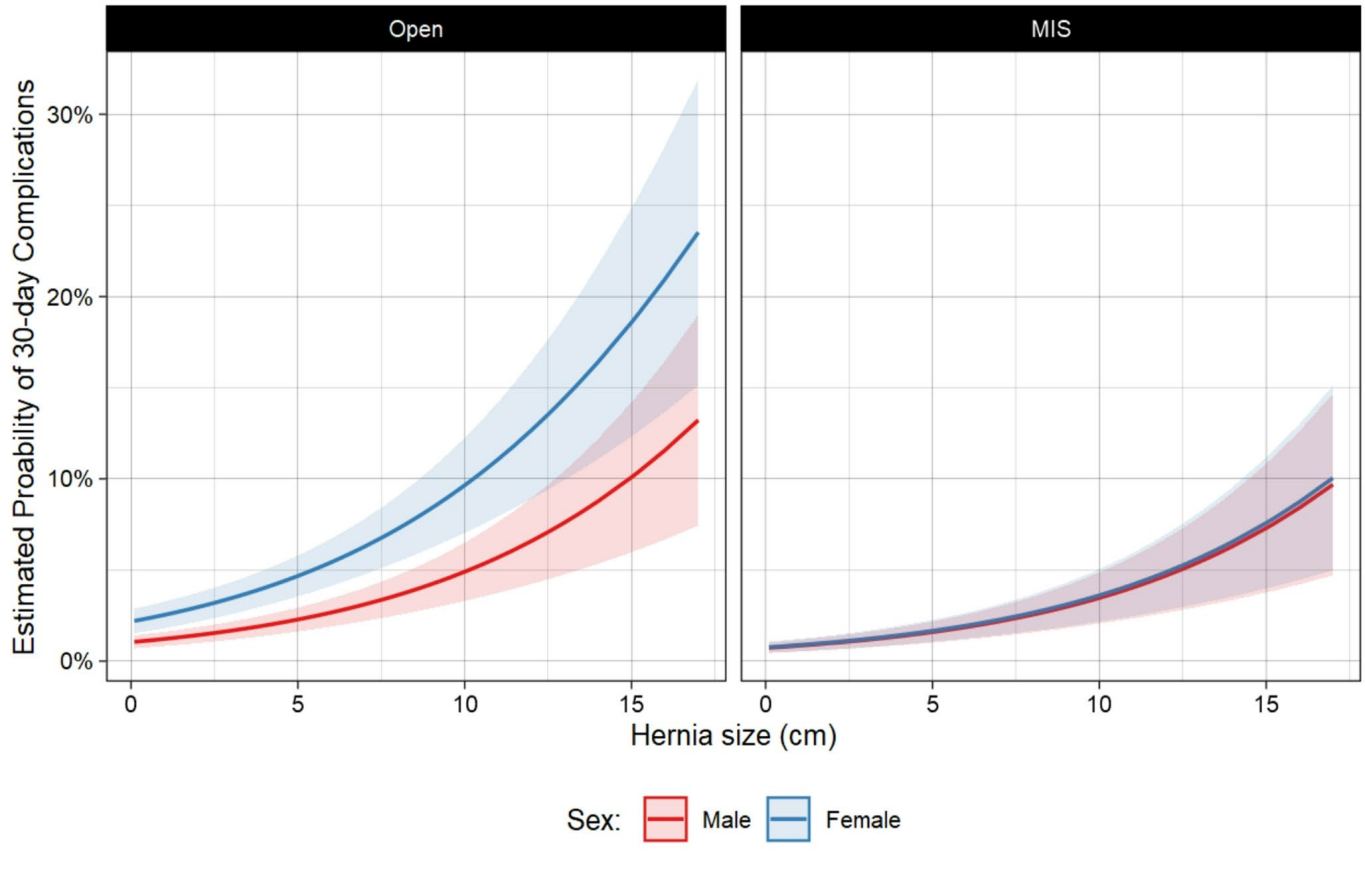
Estimated probability of 30-day complications between open and MIS VIHR in male vs. female patients

Following open VIHR, female patients were estimated to have 4.2% (95% CI: 3.2–5.1) probability of 30-day complications, versus 2.1% (95% CI: 1.5–2.7) for male patients (Fig. 2). Following minimally invasive VIHR, female patients were estimated to have a 1.5% (95% CI: 1.0-2.1) probability of 30-day complications, versus 1.5% (95% CI: 0.9-2.0) for male patients. For both male and female patients, there was a higher risk of complications following open repair versus MIS repair. However, this risk was compounded for female patients, who were 2.7 times as likely to have complications after open repair, versus male patients being 1.4 times as likely to have complications after open repair.

In addition, larger hernias (per cm aOR = 1.2, 95% CI: 1.1–1.2; *p* < 0.001), morbid obesity (aOR = 1.4, 95% CI: 1.0-1.9; *p* = 0.04), hypertension (aOR = 1.4, 95% CI: 1.0-1.9; *p* = 0.04), history of deep vein thrombosis (aOR = 2.0; 95% CI: 1.3–3.1; *p* = 0.003) and recurrent hernia repair (aOR = 1.5, 95% CI: 1.1-2.0; *p* = 0.01), were all associated with a higher likelihood of 30-day complications. Complete model specifications are available in Table 4.

**Table 4:** Complete model specifications of logistic regression model for adjusted probability of 30-day complications

	Adjusted Odds Ratio (aOR)	95% CI	*p*-value
Female Sex (ref: male)	2.13	(1.52, 2.99)	0.0000
Surgical approach, MIS (ref: open)	0.69	(0.45, 1.06)	0.09
Hernia defect size (per cm)	1.18	(1.13, 1.22)	0.0000
Hispanic ethnicity	0.89	(0.39, 1.99)	0.77
Black (ref: white)	1.17	(0.79, 1.74)	0.42
Other single race or multi-racial (ref: white)	1.53	(0.83, 2.85)	0.18
Non-commercial insurance (ref: commercial)	1.05	(0.79, 1.39)	0.75
Tobacco use (ref: no)	1.27	(0.93, 1.74)	0.14
BMI > 40 (ref: BMI < 40)	1.37	(0.97, 1.93)	0.07
Diabetes (ref: no diabetes)	1.11	(0.77, 1.60)	0.56
Hypertension (ref: no)	1.40	(1.02, 1.91)	0.04
Chronic condition requiring steroids (ref: no)	1.40	(0.80, 2.45)	0.24
DVT (ref: no)	1.98	(1.26, 3.13)	0.003
Sleep apnea (ref: no)	1.23	(0.90, 1.69)	0.18
COPD (ref: no)	1.21	(0.79, 1.87)	0.38
Non-umbilical hernia location (ref: umbilical hernia)	1.12	(0.84, 1.48)	0.44
Previous hernia repair (ref: no)	1.46	(1.08, 1.98)	0.014
Mesh use (ref: no)	0.97	(0.66, 1.42)	0.864
ASA Class (2 vs. 1)	1.71	(0.53, 5.46)	0.368
ASA Class (3 vs. 1)	2.75	(0.84, 8.96)	0.094
ASA Class (4 vs. 1)	2.91	(0.74, 11.39)	0.126
Sex (female): Surgical approach (MIS)	0.49	(0.28, 0.86)	0.013

### Differential effect in typical sized hernias

**Fig. 3:**
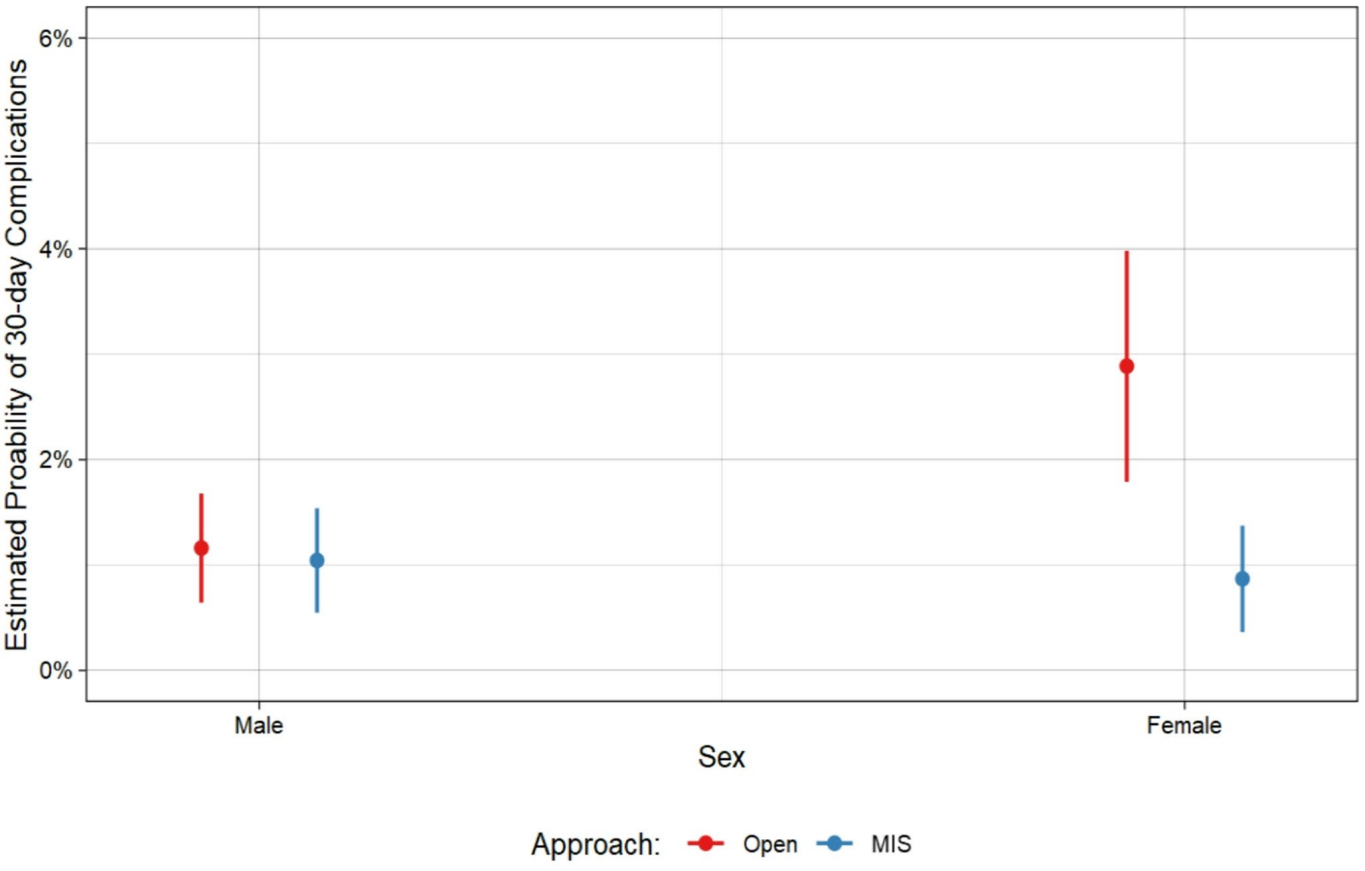
Estimated probability of 30-day complications by sex and surgical approach for typical hernias (hernias in the 25th -75th percentile, measuring 1.5–4 cm) (*p* < 0.05).

A sensitivity analysis was performed using the multivariable logistics mixed effect model with the same covariates but only included patients with hernia defects between 1.5 cm and 4 cm (the 25th to 75th percentile of hernia sizes in the sample). There were 6349 total hernia repairs ranging from 1.5 to 4 cm. The model demonstrated a similar, significant interaction effect between surgical approach and sex (*p* < 0.05), illustrated in Fig. 3, in which female patients undergoing an open repair of a typical sized VIHR had 2.9 aOR of 30-day complications (95% CI: 1.8-4.0, *p* = 0.01).

### Surgeon-specific MIS volume

A second sensitivity analysis was performed to evaluate the relationship between surgeon-specific proportional MIS volume and 30-day complications by sex. With the exclusion of surgeons with less than 10 total cases, 269 unique surgeons performing a total of 9817 cases were included. The proportion of MIS volume out of total hernia volume (open + MIS) was stratified into terciles: 90 surgeons were in the low MIS volume tercile, 89 surgeons in the middle volume tercile, and 90 surgeons in the high volume tercile. Controlling for the same set of covariates as specified above in Table 4, the interaction effect between surgical approach and sex persisted even after controlling for surgeon volume (*p* < 0.05). However, there was no difference in 30-day complication rates by sex across proportional MIS volume tertiles (*p* > 0.05).

### Sub-analysis: laparoscopic vs. robotic repairs

**Fig. 4:**
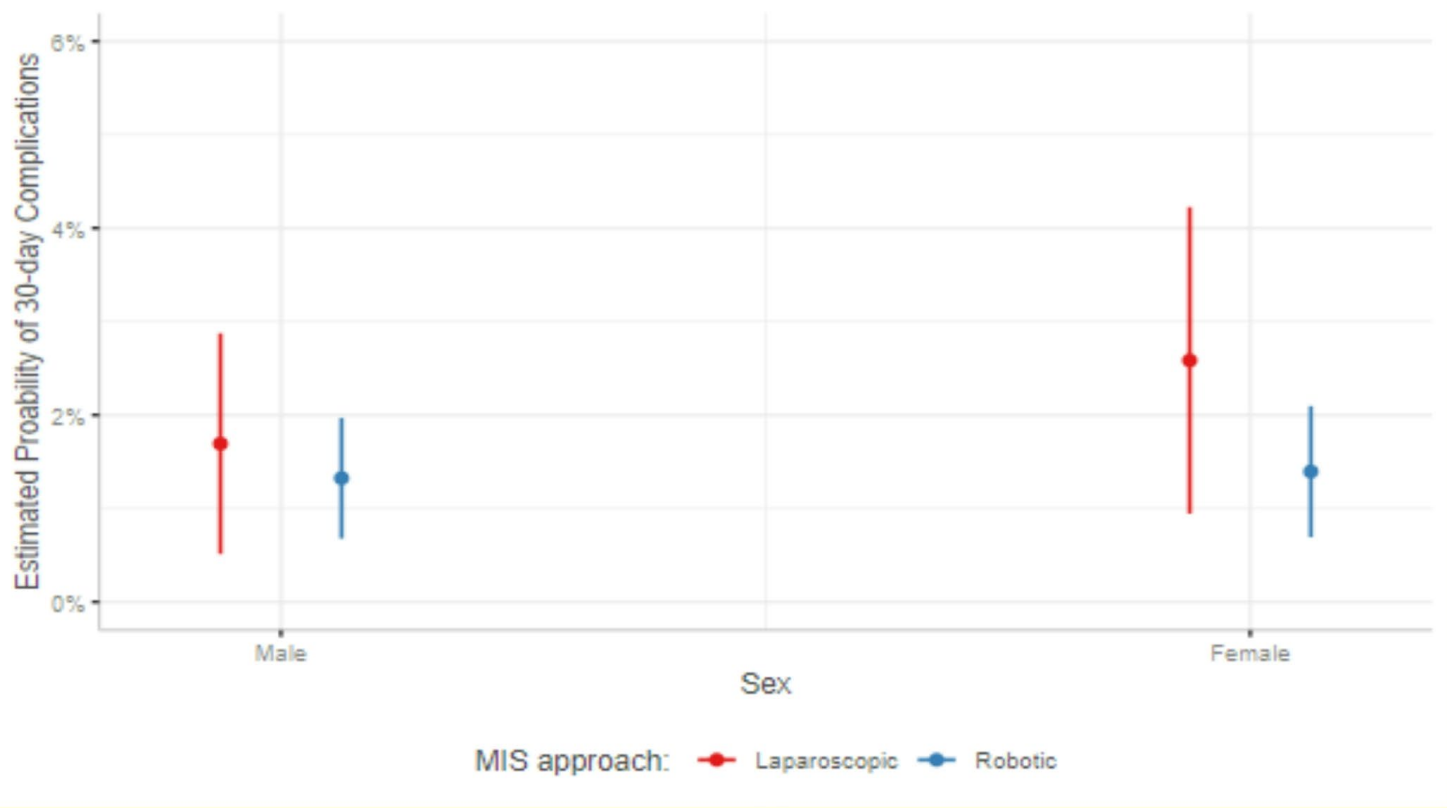
Estimated probability of 30-day complications between laparoscopic and robotic VIHR in male vs. female patients (*p* > 0.05)

A sub-analysis was performed to evaluate whether sex-based differences in 30-day complications existed between laparoscopic and robotic repairs. With the exclusion of open repairs, this analysis included 4580 MIS repairs: 1078 (23.5%) laparoscopic repairs and 3502 (76.5%) robotic repairs. In total, 75 persons (1.6%) who underwent an MIS repair experienced a 30-day complication; of these, 22 (29.3%) occurred after laparoscopic repair, and 53 (70.7%) occurred after robotic repair (*p* = 0.27). Compared to laparoscopic repair, persons who underwent robotic repair were more often Black, had larger hernia defects (3.0 cm vs. 2.3 cm, *p* < 0.001), and received mesh intraoperatively (98.0% vs. 94.6%, *p* < 0.001). A complete comparison of the cohorts by MIS repair type are available in Table 5. On multivariable logistic regression, there was no difference in 30-day complications by sex and MIS repair type (*p* > 0.05) (Fig. 4).

**Table 5:** Cohort demographics by MIS repair type

	All MIS (*n* = 4580)	Laparoscopic (*n* = 1078)	Robotic (*n* = 3502)	*p*-value
Any 30-day complication, n(%)	75 (1.6)	22 (29.3)	53 (70.7)	*p* = 0.27
Age, median (IQR)	55.0 (44–65)	56.0 (44–64)	55 (44–65)	*p* = 0.65
Race/Ethnicity, n(%)				*p* = 0.03
White	3745 (81.8)	910 (84.4)	2835 (81)	
Black	603 (13.2)	118 (10.9)	485 (13.8)	
Other or multi-racial	232 (5.1)	50 (4.6)	182 (5.2)	
Non-commercial insurance, n(%)	2745 (59.9)	657 (60.9)	2088 (59.6)	*p* = 0.46
Tobacco use, n(%)	896 (19.6)	217 (20.1)	679 (19.4)	*p* = 0.60
BMI > 40, n(%)	742 (16.2)	160 (14.8)	582 (16.6)	*p* = 0.17
ASA 3–4, n(%)	2138 (46.7)	479 (44.4)	1649 (47.4)	*p* = 0.14
Hernia defect width, cm, median (SD)	3.0 (2.5)	2.3 (2.5)	3.0 (2.5)	*p* < 0.001
Mesh use, n(%)	4453 (97.2)	1020 (94.6)	3433 (98.0)	*p* < 0.001
Mesh location, n(%)				*p* < 0.001
Onlay	606 (13.2)	181 (16.8)	425 (12.1)	
Inlay	152 (3.3)	87 (8.1)	65 (1.9)	
Sublay	3477 (75.9)	667 (61.9)	2810 (80.2)	
Unknown	218 (4.8)	85 (7.9)	133 (3.8)	
No mesh	127 (2.8)	58 (5.4)	69 (2.0)	
Previous hernia repair, n(%)	740 (16.2)	183 (17.0)	557 (15.9)	*p* = 0.42
Hernia location, n(%)				*p* = 0.02
Umbilical	2925 (63.9)	720 (66.8)	2205 (63.0)	
Epigastric	1040 (22.7)	247 (22.9)	793 (22.6)	
Infraumbilical	267 (5.8)	48 (4.5)	219 (6.3)	
Suprapubic	64 (1.4)	12 (1.1)	52 (1.5)	
No midline component	284 (6.2)	51 (4.7)	233 (6.7)	

## Discussion

In this study that evaluated the effects of surgical approach on short-term outcomes following VIHR, there were three key findings. First, female patients were more likely than male patients to have 30-day complications, regardless of operative approach. Second, as has been shown in prior studies, open VIHR was associated with higher probability of 30-day complications for both male and female patients. Third, an MIS approach narrowed the disparity in risks of postoperative complications between male and female patients. Overall, these findings suggest that MIS approaches should be preferentially pursued in female patients when technically feasible.

The results of this study contribute to a growing body of literature around sex-based differences in hernia surgery outcomes. Prior studies have reported that female patients undergoing ventral hernia repair have higher rates of postoperative complications and are more likely to undergo complex repairs [5, 8, 10, 12, 16]. Our study aligns with these findings and expands upon the data by demonstrating that MIS approaches not only reduce overall complication rates for female patients but also attenuate the observed sex-based disparities in complications. Notably, we found no difference by sex in 30-day complication rates between laparoscopic and robotic VIHR, indicating that MIS approaches are beneficial for female patients regardless of the specific modality used. As robotic technology becomes more widely available, it may offer an increased opportunity for MIS-based repairs in both male and female patients. Robotic surgery has seen a dramatic rise in utilization, and it may help shift VIHR away from open techniques when feasible [28–30].

As we aim to identify levers to reduce sex-based disparities in hernia surgery outcomes, our study isolates two potential targets: operative approach and preoperative optimization. Regarding operative approach, our data indicates that MIS approaches demonstrated a lower risk of postoperative complications for female patients, as compared to open approaches. In our study, we found that open repairs were associated with a 4.2% probability of 30-day complications for female patients, versus a 1.5% probability after MIS repairs. Male patients had lower probability of complications across both surgical approaches, but the complication gap between open and MIS repair was smaller, with 2.1% risk following open repair and 1.5% risk following MIS repair. As demonstrated in this comparative data, open repairs were associated with a compounded risk of complications for female patients, whereas MIS repairs mitigated the disparity in risk between male and female patients. While the outcome gap was not fully eliminated between the sexes, this data suggests that MIS repairs should be prioritized in female patients whenever possible. If the relative benefits of MIS approaches for VIHR in female patients are reproduced with long-term clinical outcomes and with patient-reported outcomes (e.g., chronic pain, decision regret), then incorporating this recommendation into hernia guidelines should follow.

The second area of potential intervention is preoperative optimization. In our study, female patients had a higher baseline risk profile, particularly with respect to well-recognized modifiable risk factors, such as active tobacco use and obesity [31, 32]. Female patients also had larger hernia defects, and they more commonly underwent recurrent hernia repairs. The reasons for this higher baseline risk are unknown, but it raises concern that female patients were less likely to undergo preoperative optimization for smoking cessation and weight loss. The link between optimization and outcomes has been well-demonstrated, with previous work showing that lack of optimization resulted in more emergency department visits and postoperative complications [33, 34]. Alternatively, attempts at optimization—and the time associated with optimization—may ultimately generate the larger hernia defects that female patients demonstrate at the time of repair [35]. Female patients, as well as patients with more socioeconomic distress, may be preferentially impacted by preoperative optimization protocols, and consideration of these cutoffs as potential sources of access disparities should be taken into account [36]. Ultimately, ensuring that female patients receive appropriate preoperative optimization as well as timely surgical intervention can reduce inequities in surgical outcomes.

This work should be interpreted within the context of several important limitations. This study focuses only on short-term (30-day) clinical complications. For hernia surgery, major outcomes of interest include long-term clinical and patient-reported outcomes. While we did not evaluate long-term outcomes in this study, understanding short-term complications is important foundational work, as data shows that short-term complications (e.g., surgical site infection, readmission) have long-term effects. Data shows that short-term complications increase the risk of long-term reoperative recurrence or chronic pain [37]. Future studies that evaluate the impact of surgical approach on long-term clinical complications between sexes would be important. Similarly, given that the procedure of interest is a quality-of-life operation, evaluating whether operative approach differentially impacts patient-reported outcomes (e.g., chronic pain or decision regret) would be of interest as we continue to tailor hernia surgery care to patient-reported outcomes. Ultimately, the exact reasons for the higher complication rates observed among female patients, regardless of operative approach, are unknown. As this was a retrospective study rather than a blinded prospective study, the differential effect of operative approach on postoperative complications may be attributable to the higher risk profile of those female patients selected to undergo open repair. Alternatively, other unmeasured confounders (e.g., repair of diastasis recti, panniculectomy, or sex-specific anatomic/morphomic factors) may differentially impact the risk of complications for female patients.

## Conclusion

At baseline, female patients were found to have higher preoperative risk profiles, including obesity, smoking, larger hernia defects, and a higher incidence of recurrent repairs. After adjusting for this increased preoperative risk, female sex remained an independent risk factor for 30-day complications following VIHR, regardless of operative approach. However, our data suggests that MIS approaches narrow the outcome disparity between male and female patients, though the gap is not completely erased. These results suggest that MIS approaches, whether laparoscopic or robotic, should be prioritized in female patients undergoing elective VIHR to improve postoperative outcomes for females and promote equity in surgical care.
